# Supplementary material for: Neutralizing human monoclonal antibodies to poliovirus map to the receptor binding site
Source: Nat Commun. 2026 Jan 2;17:1512. doi: 10.1038/s41467-025-68226-x (PMC12891506; doi:10.1038/s41467-025-68226-x)
Supplement: Supplementary file 1 — Supplementary Information [file 41467_2025_68226_MOESM1_ESM.pdf]

Figure S1: cryoEM particles and local resolution for PV-FAb complexes A) Representative motion corrected micrographs, demonstrating FAb binding to virus capsids. **B)** 2D classes for final cryoEM reconstructions **C)** Gold-standard FSC curves for PV-Fab complex EM maps. **D)** Local resolution mapping of PV-FAb complex maps, with coloring measured in angstroms according to key (right). The strongest density was shown for the virus capsid with the weakest density shown for the constant domain and hinge region of the FAb. **E)** Quality of the protein model built into representative EM map density (grey). Shown are residues 158-164 of VP2 (green stick) with additional coloring by heteroatom.

Supplemental Figures

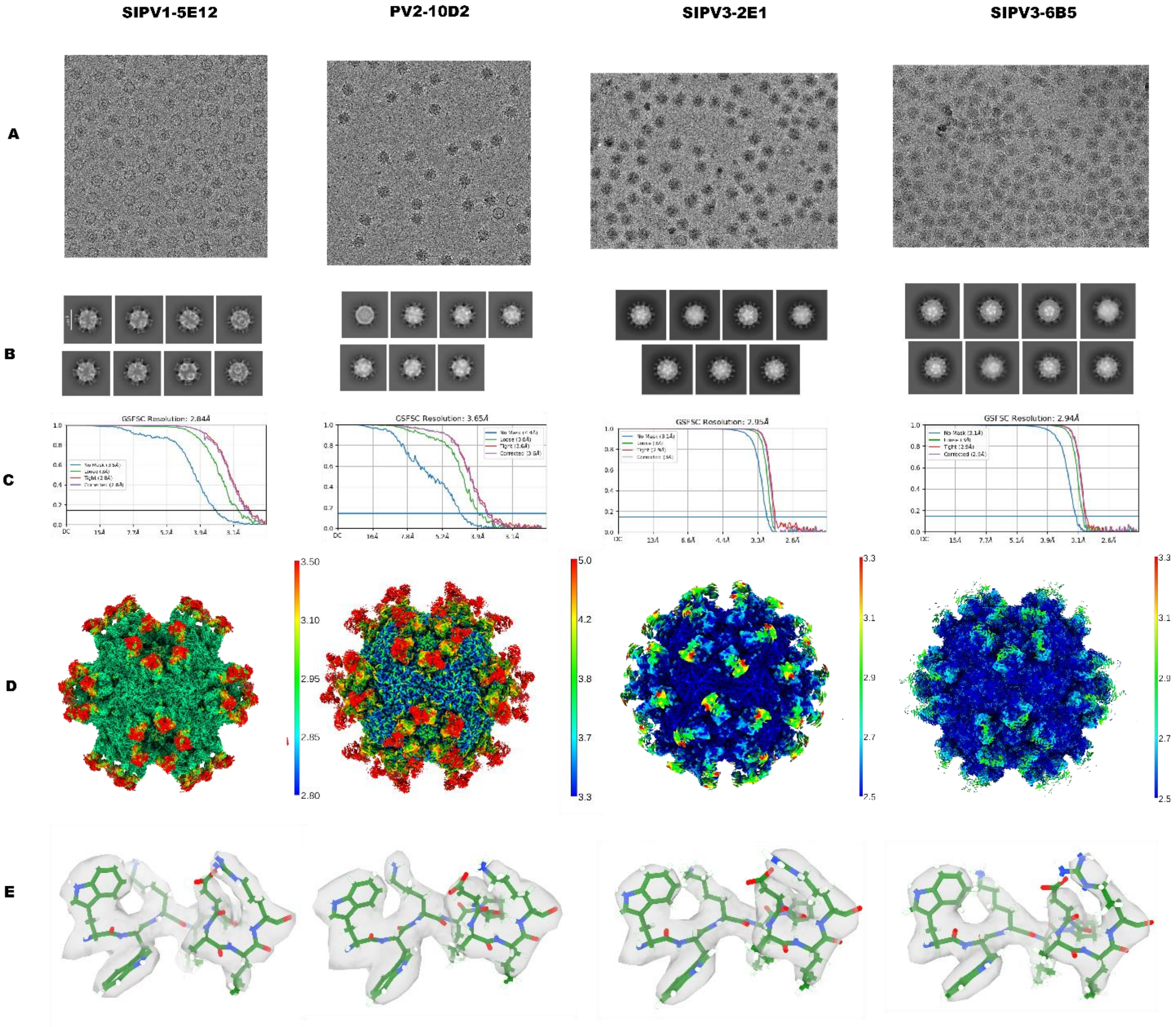

Figure S2: Comparative binding footprints between Five-fold binding FAbs and the poliovirus receptor. Poliovirus capsid proteins are represented as surface-rendered chains, with VP1, VP2, VP3, and VP4 colored blue, green, red, and yellow, respectively. Heavy chains (goldenrod) and light chains (dark gray) are represented as ribbons, as is the poliovirus receptor (magenta)[1,2].

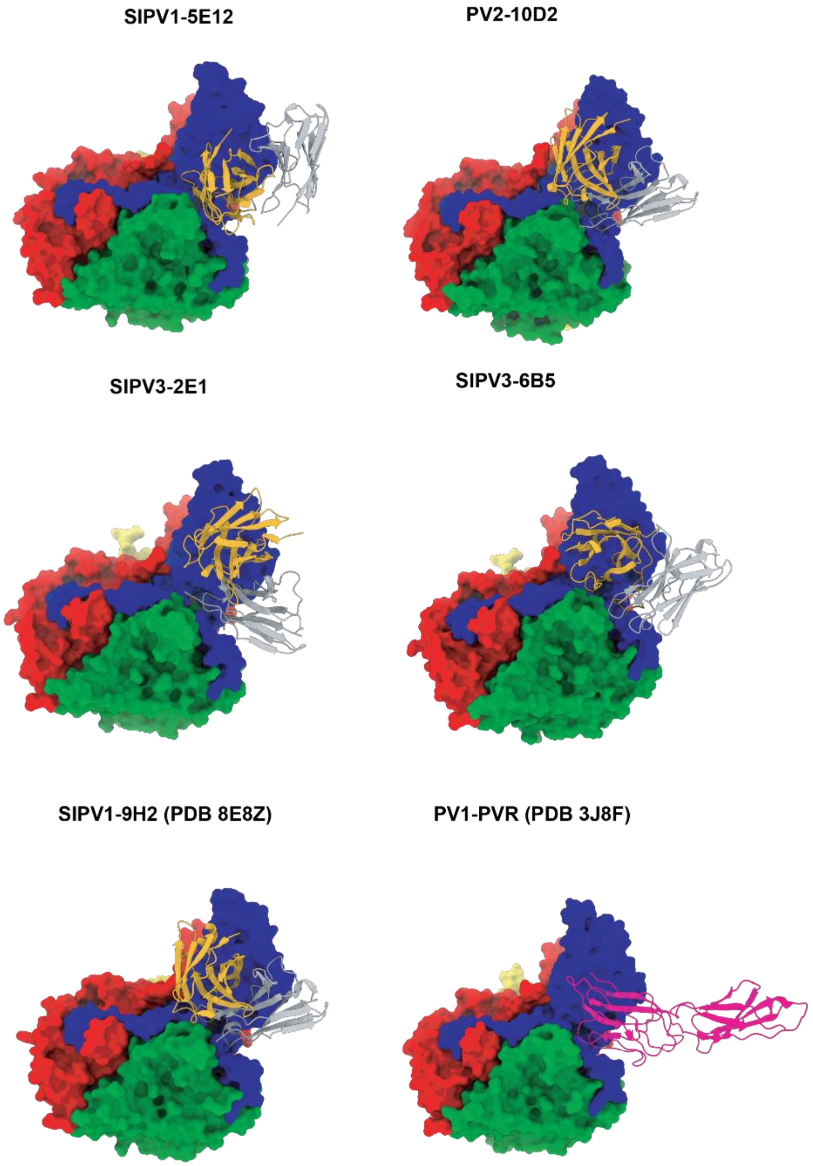

Figure S3: FAb binding induces local conformational change near binding interface. Per-residue C-alpha RMSD values (colored in angstroms) on the virus protomer (gray) compared to representative apo PV capsid crystal structures (PDB ID 1HXS, 1EAH, and 1PVC for type 1, type 2, and type 3 poliovirus, respectively)[3-5]

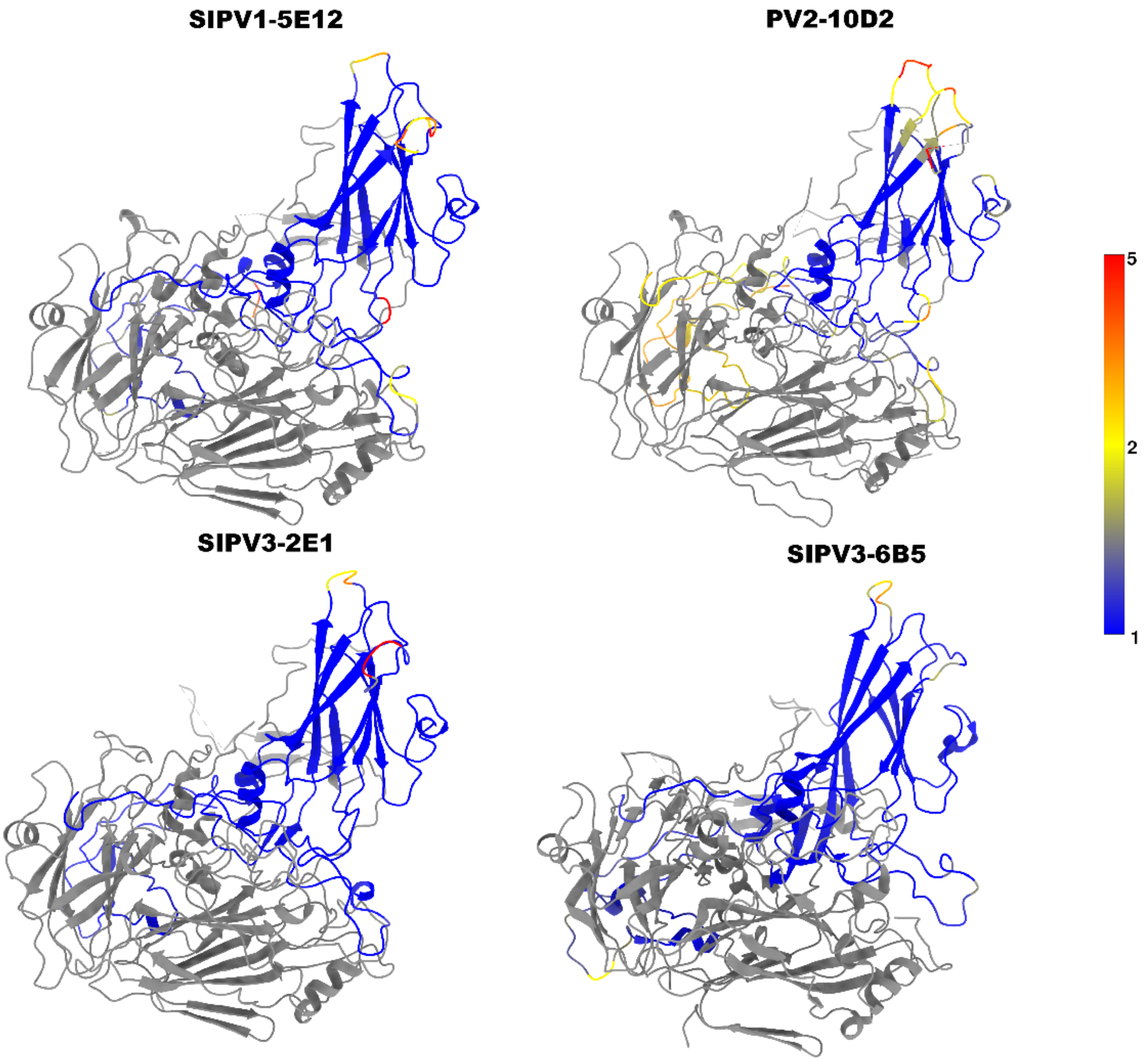

Figure S4: VP1 GH Loop of PV-FAb complexes are in "doorstop-up" conformation. The VP1 GH loop (residues 230-237) are in the doorstop-up conformation in the SIPV1-5E12 complex (blue). This conformation is observed in PV A-particles (green) and PVR-bound PV (orange-red). Conversely, Apo-PV (Magenta) is in the doorstop-down conformation. The doorstop-up conformation has been shown previously to be key for release of pocket factor and particle maturation in PV capsids[2,3,6]

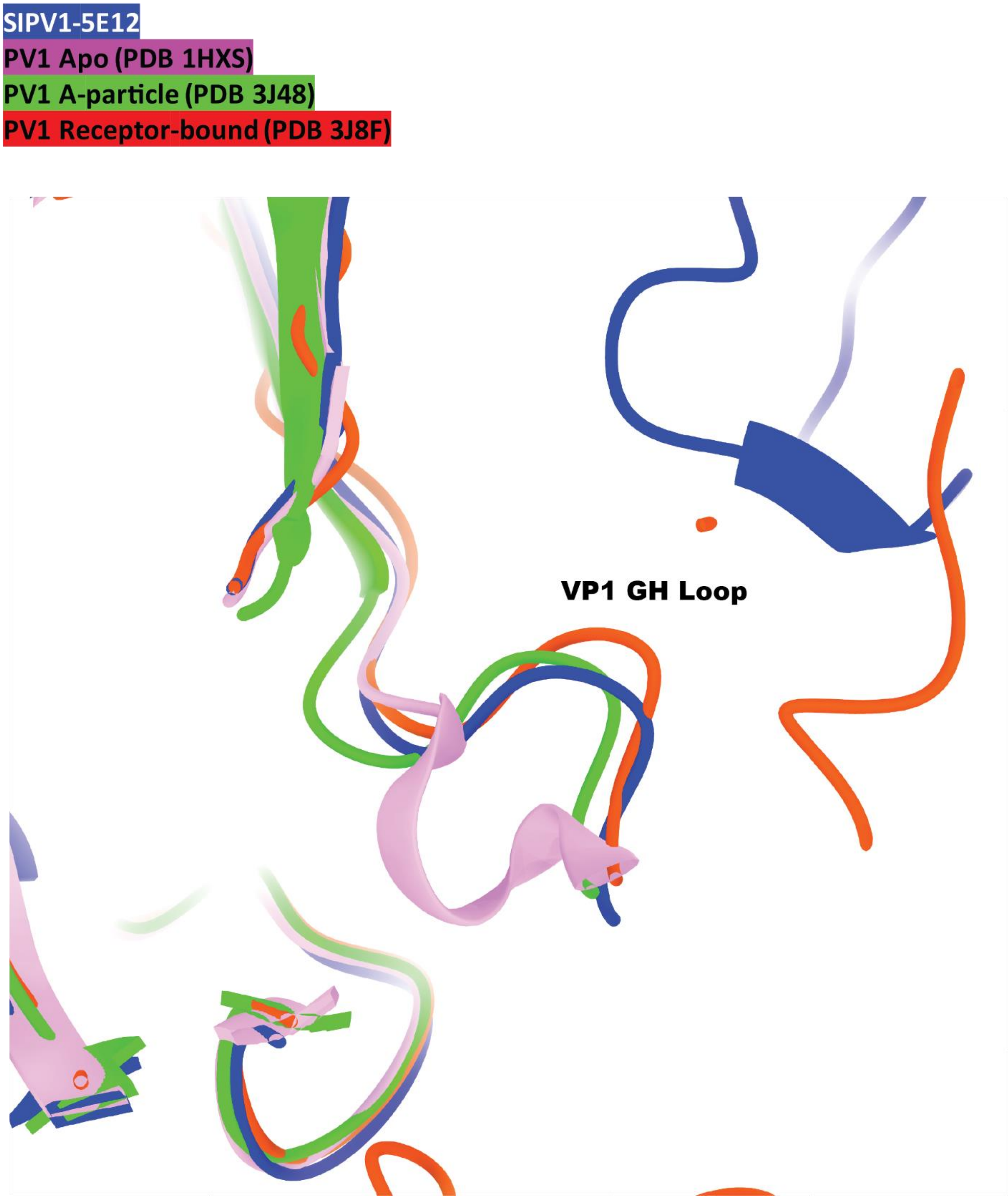

Figure S5: Five-fold binding FAb footprints on PV capsids. Roadmap projections[7] showing individual FAb binding footprints to PV capsids for 5E12, 10D2, 2E1, and 6B5 (green, blue, red, yellow, respectively as labeled).

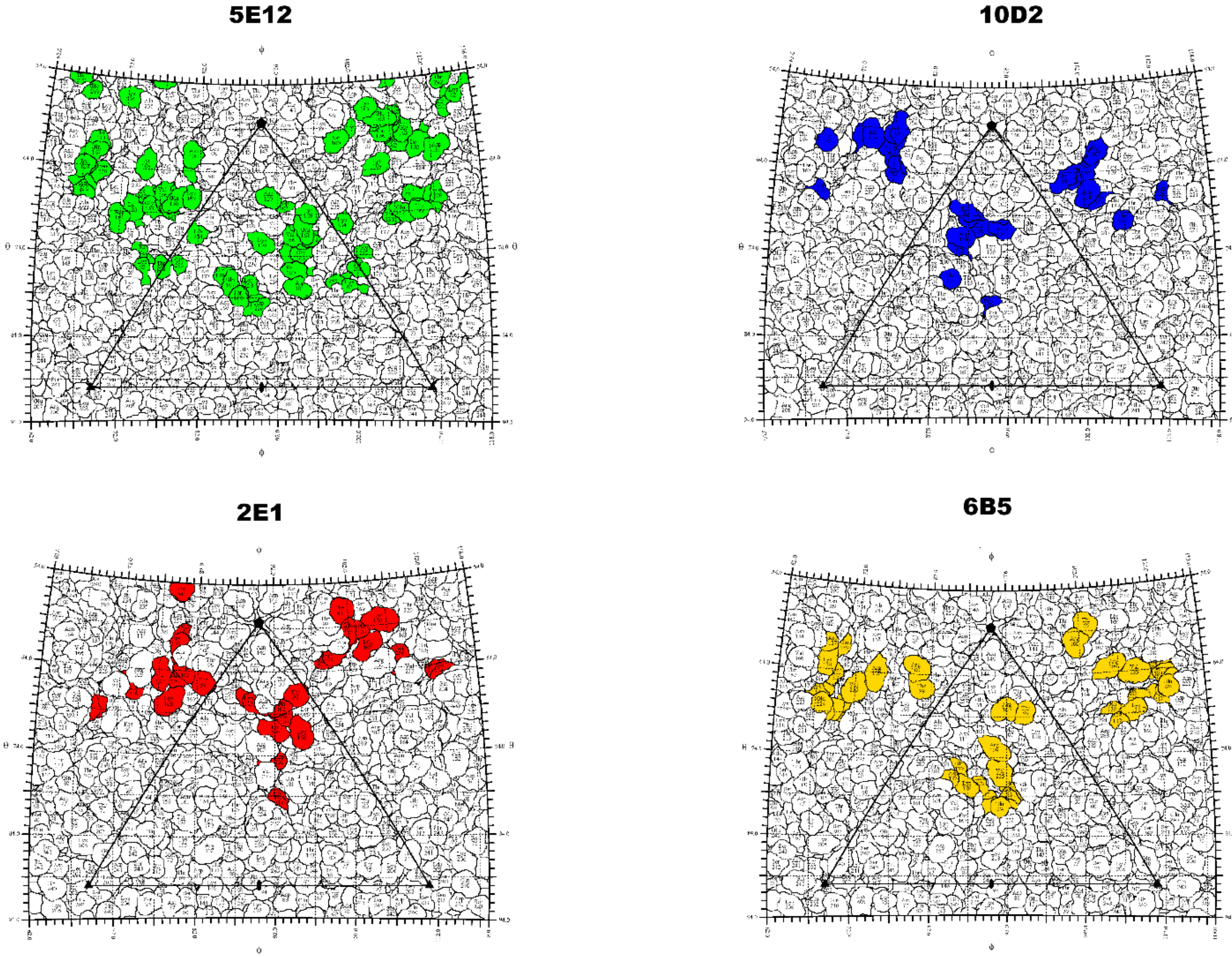

Figure S6: Pocket factor is retained in all PV-FAb complexes. EM map density (grey) corresponding to that of pocket factor, modeled as palmitic acid in orange, with additional coloring by heteroatom. Coloring of capsid and FAb chains corresponds to color key as shown (right).

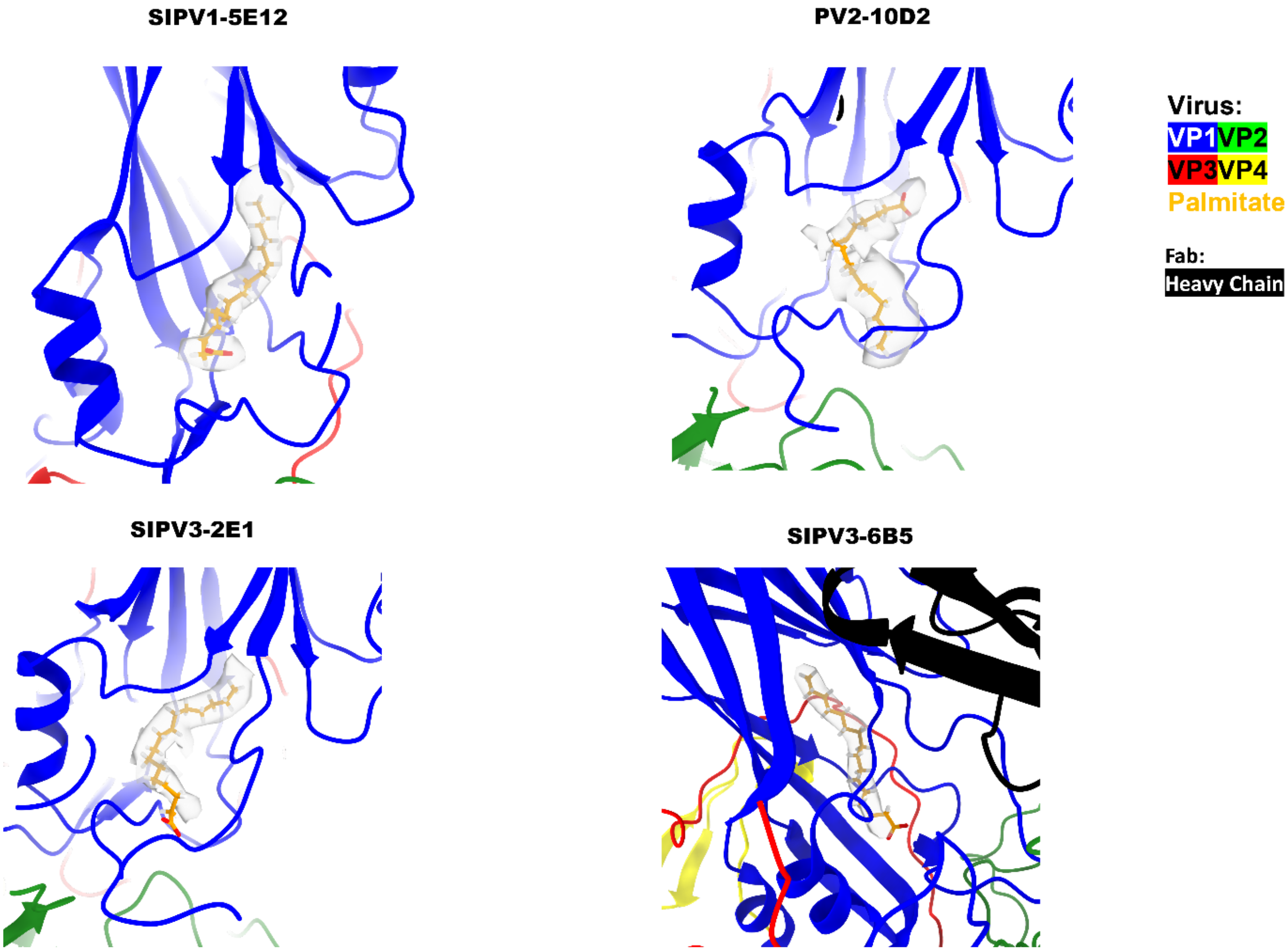

Figure S7: Five-fold binding FAbs overlap with 9H2 footprint. Roadmaps showing comparisons of common contacts (gray) between five-fold binding FAbs and 9H2 on capsids (see color key above).

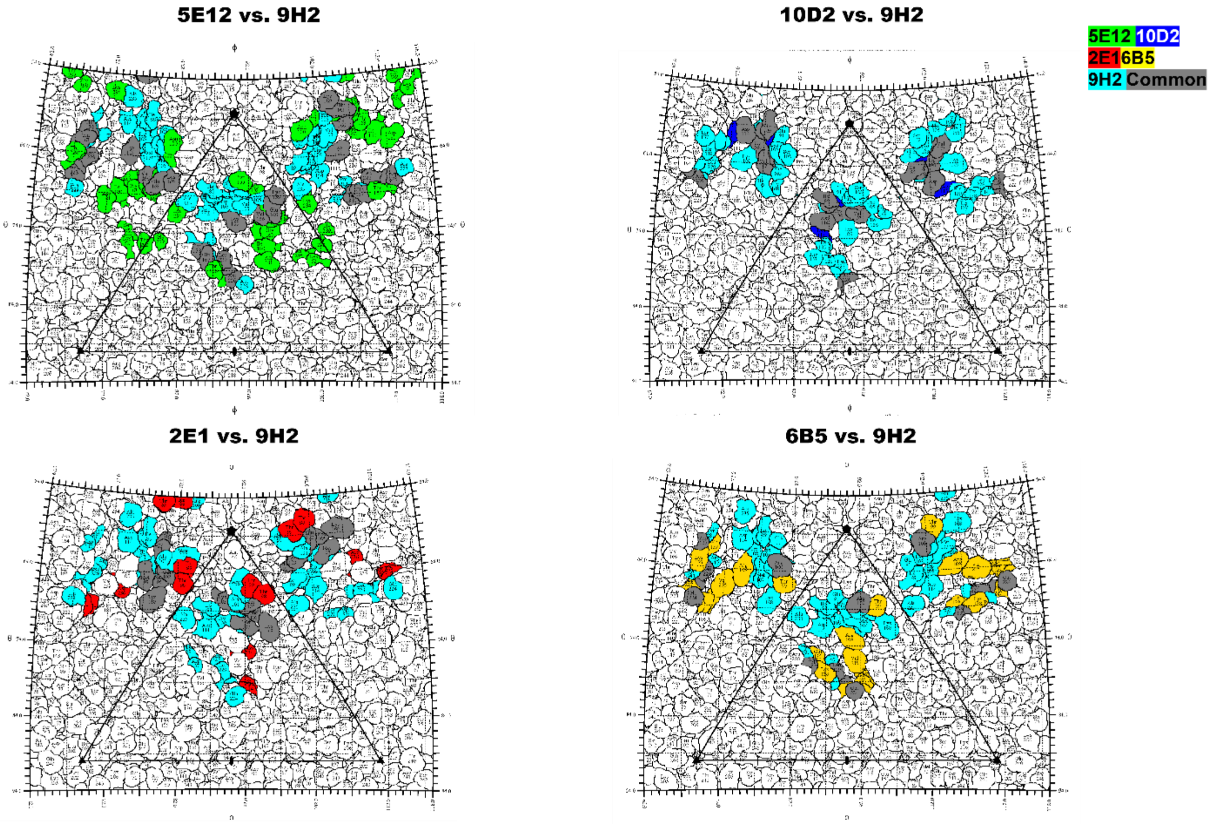

Figure S8: Comparative modes of receptor engagement between enteroviruses Capsid proteins VP1-4 are shown as surface-rendered models, colored blue, green, red, and yellow for VP1, VP2, VP3, and VP4, respectively. Receptor molecules as shown as ribbons and colored magenta. Protein models are identified by PDB code, as shown[2,8-13]

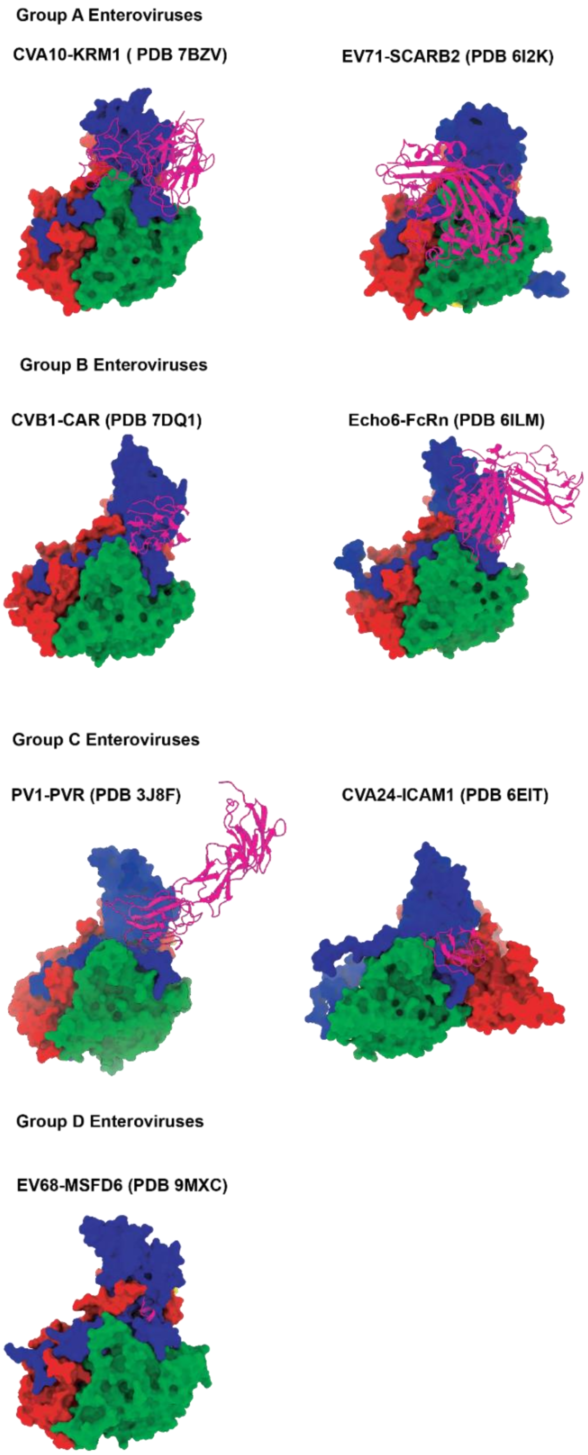

Figure S9: Reconstruction workflow for SIPV1-5E12 complex. The flow chart presents the data processing pipeline for the SIPV1-5E12 complex, showing pre-processing, 2D classification, icosahedral reconstruction, and final refinements the icosahedral map.

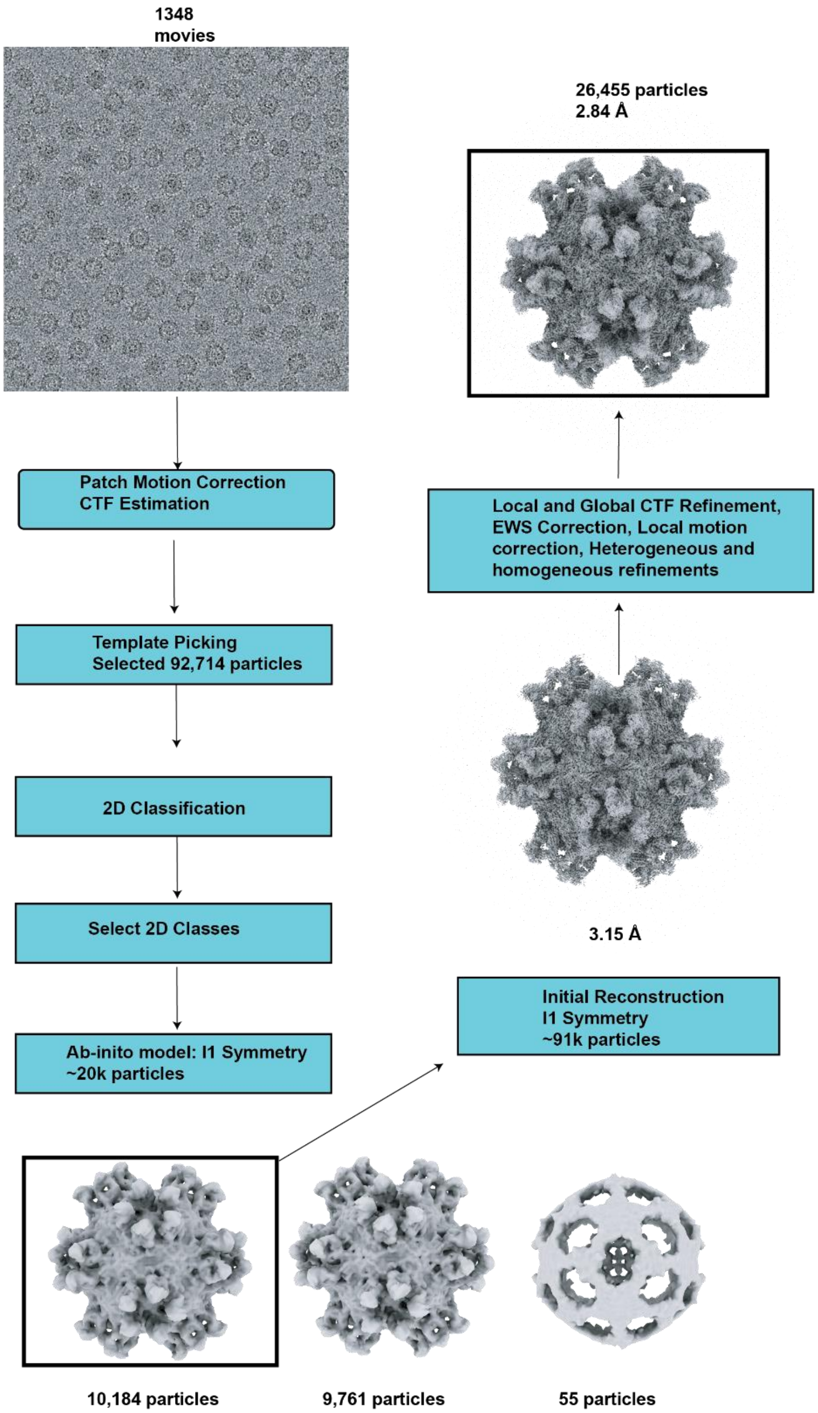

Figure S10: Reconstruction workflow for PV2-10D2 complex. The flow chart presents the data processing pipeline for the PV2-10D2 complex, showing pre-processing, 2D classification, icosahedral reconstruction, and final refinements the icosahedral map.

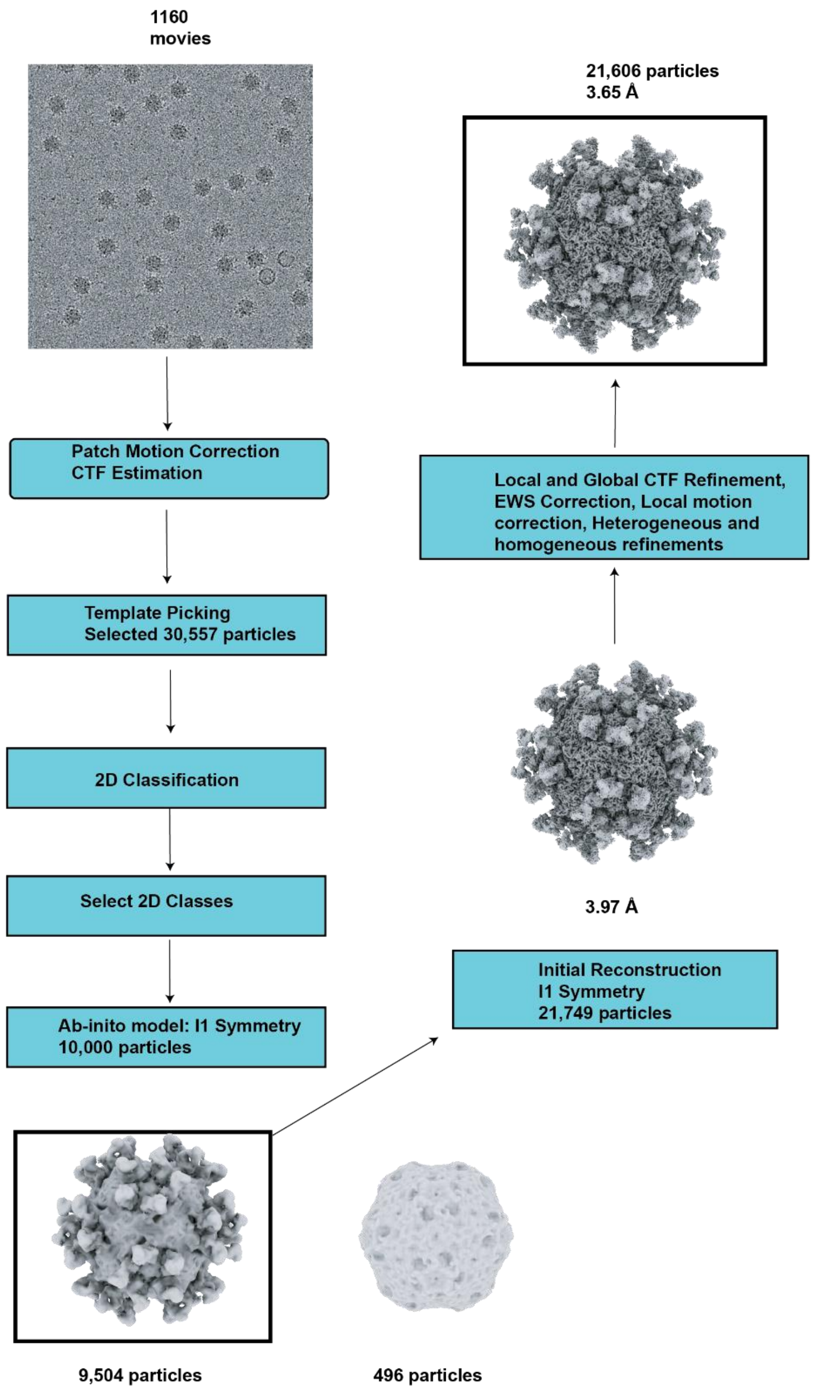

Figure S11: Reconstruction workflow for SIPV3-2E1 complex. The flow chart presents the data processing pipeline for the SIPV3-2E1 complex, showing pre-processing, 2D classification, icosahedral reconstruction, and final refinements the icosahedral map.

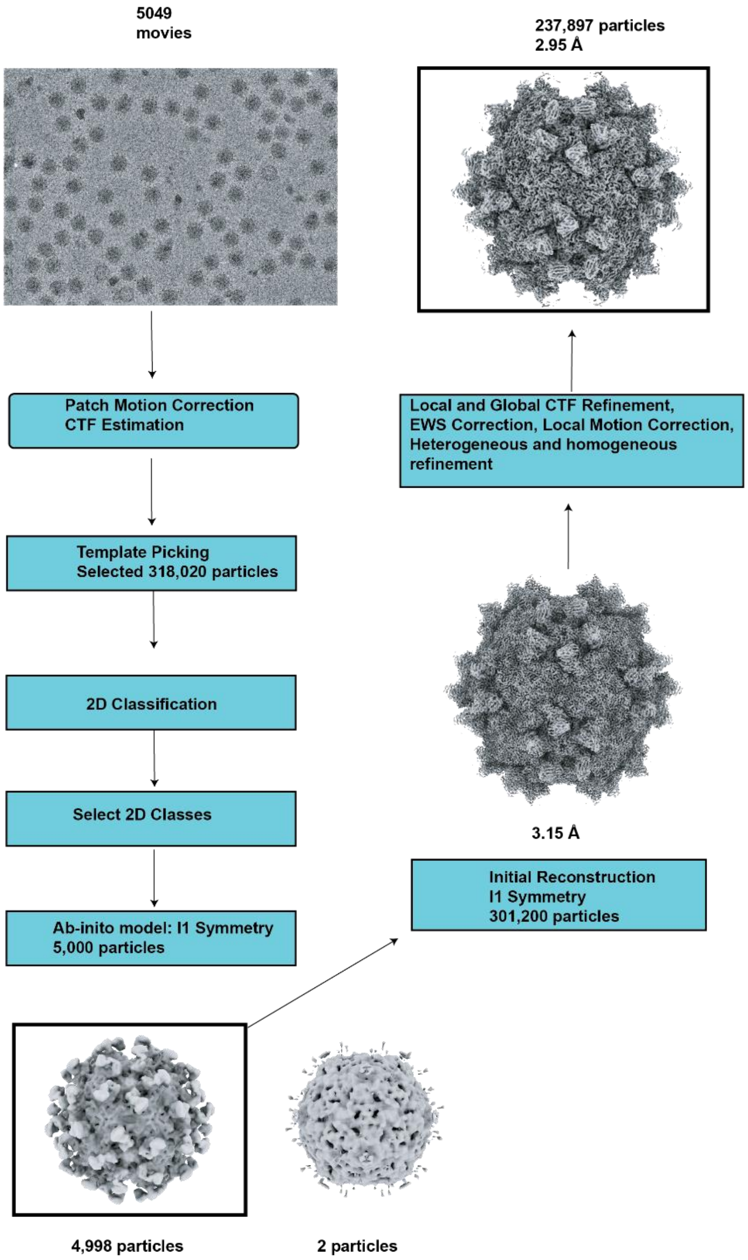

Figure S12: Reconstruction workflow for SIPV3-6B5 complex. The flow chart presents the data processing pipeline for the SIPV3-2E1 complex, showing pre-processing, 2D classification, icosahedral reconstruction, and final refinements the icosahedral map.

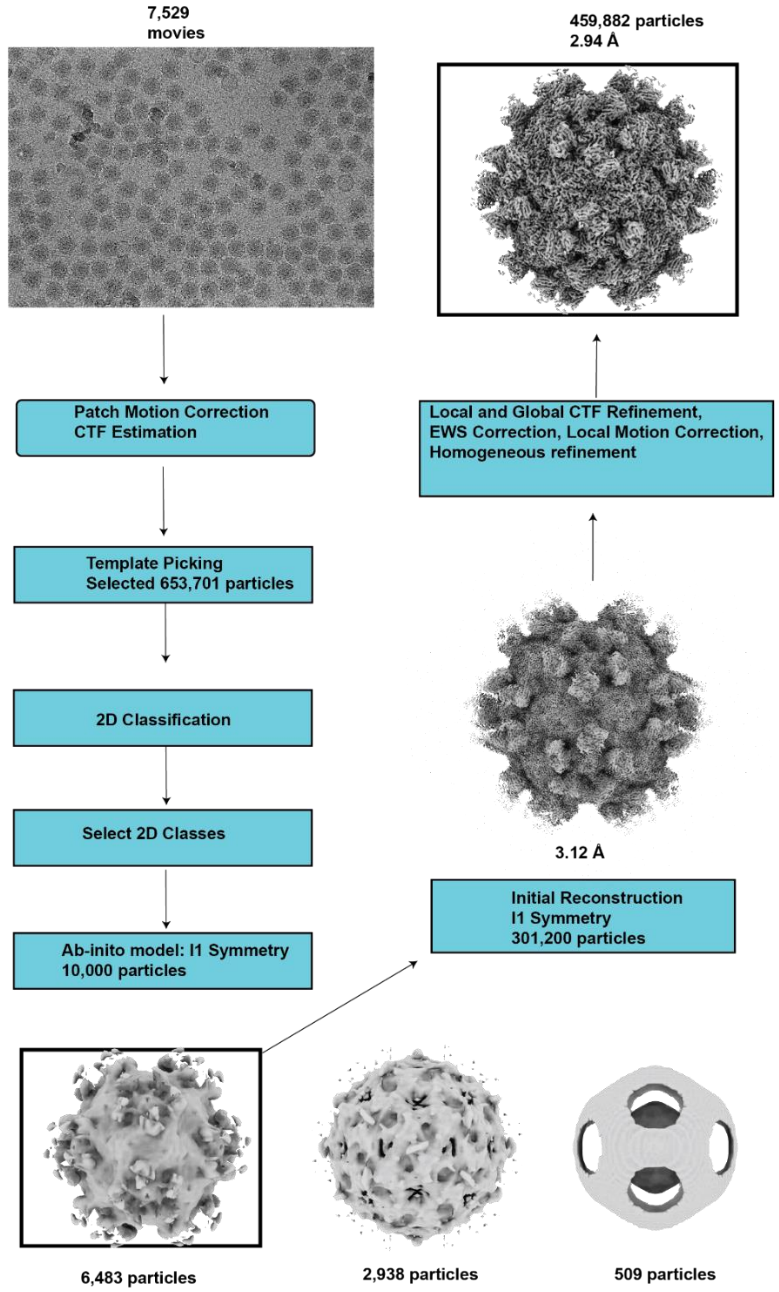

Figure S13: Sequence alignment of capsid proteins for wild-type and vaccine-strain polioviruses. ClustalOmega[14] sequence alignment of capsid proteins VP1, VP2, and VP3. UniProt accession numbers for wild-type PV sequences are P03300, P06210 and P03302 for PV1-Mahoney, PV2-Lansing, and PV3-Leon, respectively. UniProt accession numbers for Sabin PV sequences are P03301 and Q8B3S1 for SPV1 and SPV2, respectively, with P03302 used for SPV3. **A)** Sequence alignment of capsid protein VP1 **B)** Sequence alignment of capsid protein VP2 **C)** Sequence alignment of capsid protein VP3

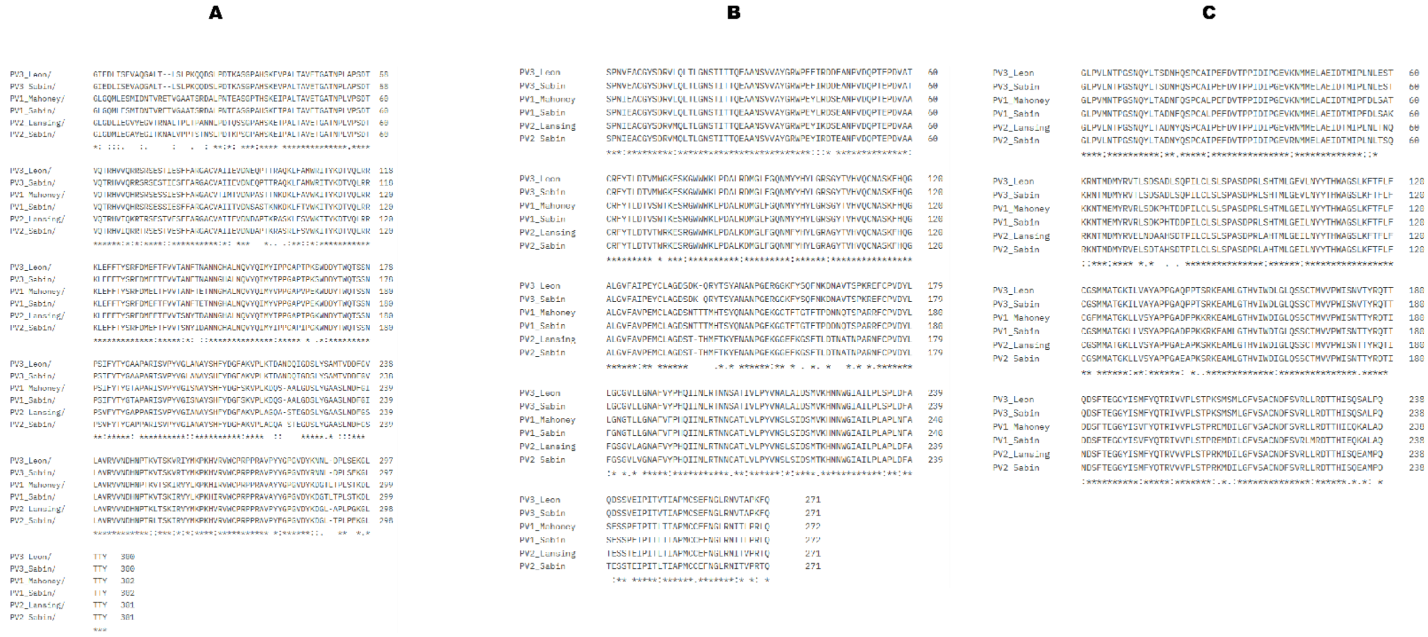

Figure S14: Five-fold binding antibodies neutralize as mAb and FAb *in-vitro*. Error bars representative of standard error of mean (SEM) for *n=3* technical replicates. **A)** Neutralization efficiency (calculated as percentage of viable wells 72 hpi) of mAbs towards either SPV1 (5E12) or SPV3 (10D2, 2E1, and 6B5) as a function of mAb concentration (in mg/mL). **B)** Neutralization efficiency (calculated as percentage of viable cells 72 hpi) of mAbs towards either SPV1 (5E12) or SPV3 (10D2, 2E1, and 6B5) as a function of FAb concentration (in mg/mL).

A

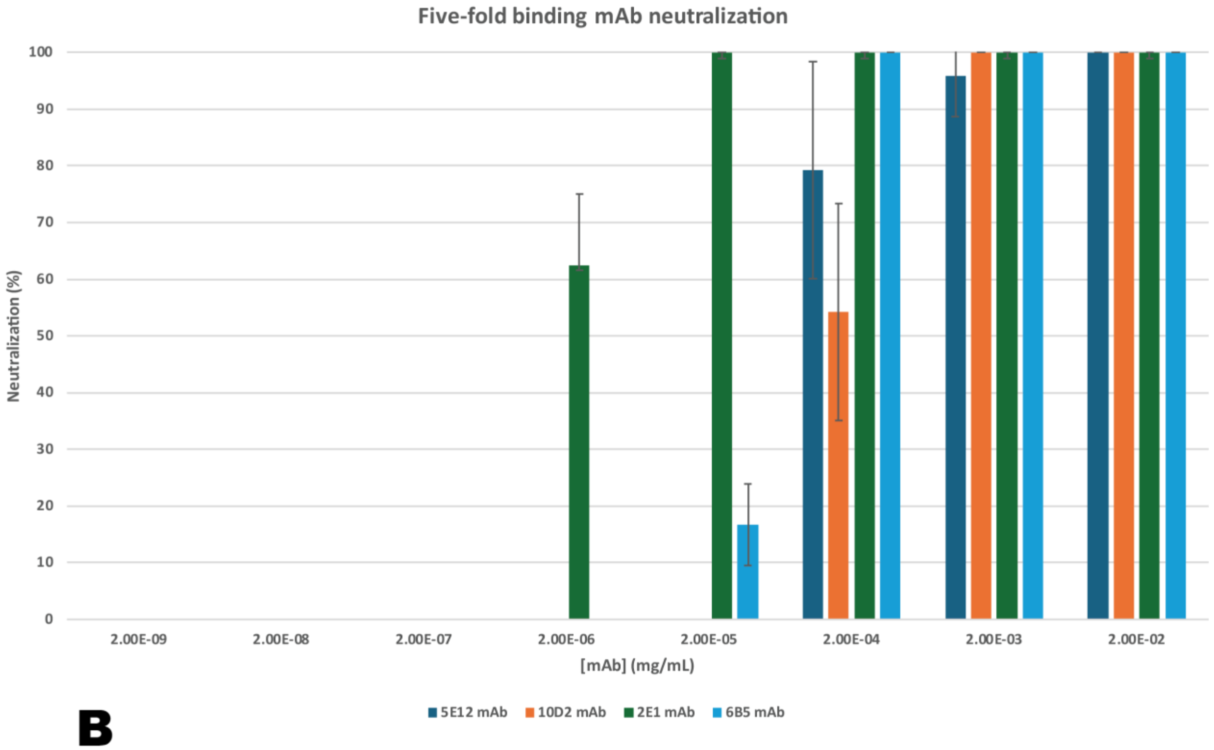

B

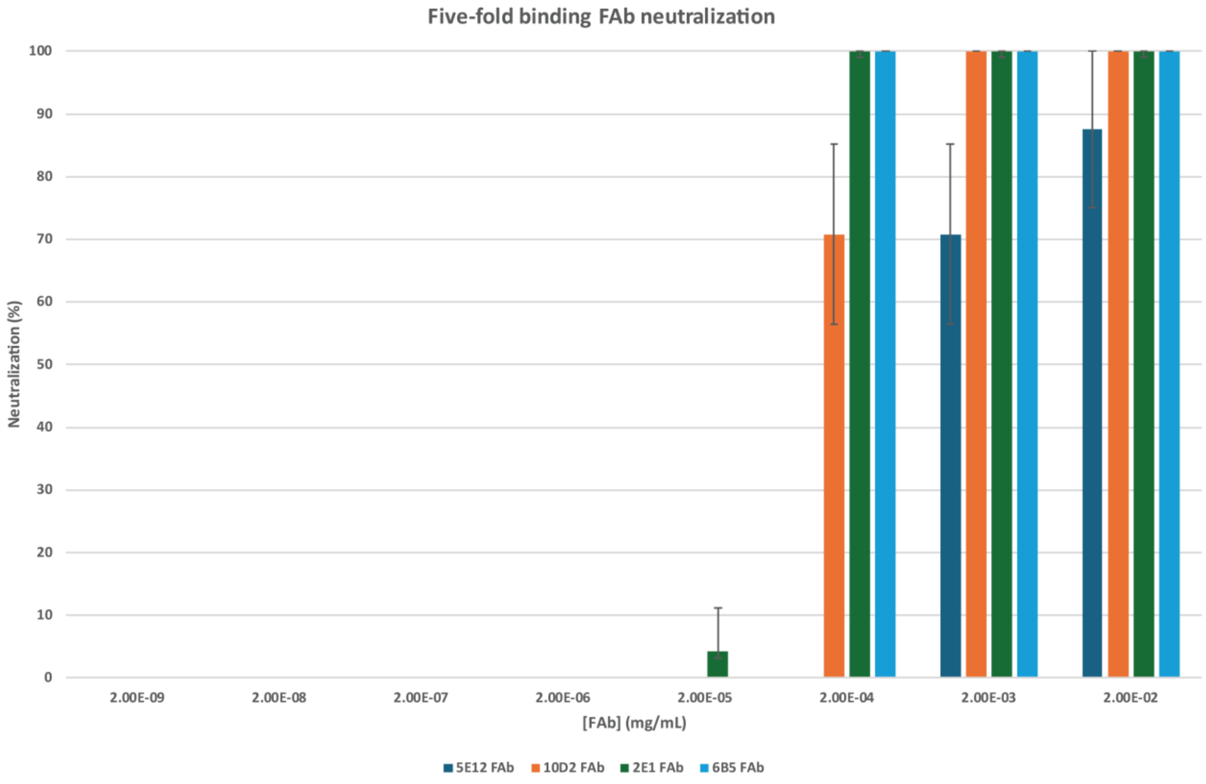

Table S1: Data collection and refinement statistics for cryoEM maps and protein models. Statistics and parameters for each PV-FAb complex, with final refinement statistics provided by Phenix comprehensive validation[15].

	SIPV1-5E12	PV2-10D2	SIPV3-2E1	SIPV3-6B5
**Magnification**	120K	120K	81K	81K
**Voltage (kV)**	200	200	300	300
**Dose-rate (e/Å^2)**	60	60	56	50
**Pixel Size (Å)**	1.3	1.3	1.1	1.1
**Defocus Range (μm)**	-0.75-2	-0.75-2	-0.5-2	-0.5-2
**Symmetry Imposed**	I1	I1	I1	I1
**Initial Micrographs**	1348	1160	5049	7529
**Final Micrographs**	1330	1043	5038	7493
**Particle Count**	26,641	21,606	237,897	459,882
**Final Resolution (Range) (Å) (FSC=0.143)**	2.84 (2.62-3.20)	3.65 (3.18-6.87)	2.95 (2.42-7.16)	2.94 (2.43-3.68)

**Model Resolution (Å) (FSC=0.143)**	2.7	3.6	2.9	2.9
**Map Sharpening B-factor**	-140.8	-174.5	-162.9	-156.6
**Non-Hydrogen Atoms**	8,347	8,275	8,368	8,353
**Protein Residues**	1065	1065	1071	1072
**Protein B-factor (Å^2)**	88.61	83.60	61.40	55.31
**Ligands**	PLM	PLM	PLM	PLM
**Ligand B-factor (Å^2)**	53.11	76.52	77.6	67.1
**Bond length RMSD (Å)**	0.003	0.002	0.003	0.003
**Bond Angle RMSD (°)**	0.542	0.585	0.461	0.545
**MolProbity Score**	1.15	1.33	1.31	1.06
**Clashscore**	1.70	2.09	2.25	1.28
**Ramachandran favored (%)**	96.47	94.95	95.55	96.88
**Ramachandran outliers (%)**	0	0.1	0	0.09
**Rotamer Outliers (%)**	0	0	0	0

Table S2: Global conformational change induced by FAb binding on PV capsids. Calculations for C-α backbone RMSD (Å) of FAb-PV models compared to apo-PV or 9H2-PV complexes, performed using MatchMaker function in UCSF Chimera[1,3-5,16]

Protein structure	PDB Model	FAb	C-α backbone RMSD (Å)
Apo PV1 Capsid	1HXS	5E12	0.814
PV1 capsid-9H2	8E8L	5E12	0.715
SIPV1 capsid-9H2	8E8Z	5E12	0.665

Protein structure	PDB Model	FAb	C-α backbone RMSD (Å)
Apo PV2 Capsid	1EAH	10D2	1.098
PV2 capsid-9H2	8E8S	10D2	1.101
SIPV2 capsid-9H2	8E8Y	5E12	1.062

Protein structure	PDB Model	FAb	C-α backbone RMSD (Å)
Apo SPV3 Capsid	1PVC	2E1	0.482
SPV3 capsid-9H2	8E8R	2E1	0.602
SIPV3 capsid-9H2	8E8X	2E1	0.509

Protein structure	PDB Model	FAb	C-α backbone RMSD (Å)
Apo SPV3 Capsid	1PVC	6B5	0.855
SPV3 capsid-9H2	8E8R	6B5	0.973
SIPV3 capsid-9H2	8E8X	6B5	0.811

Table S3: FAb contacts overlap with 9H2 binding footprint. Amino acid residues numbered by sequence position on corresponding capsid proteins

	5E12	Common	9H2
VP1	100,102,161,162, 163,264,165,167, 289,292,294,295	109,168,169,226, 227,228	87,88,89,90,101
VP2	142,168,172	138,167	139,140
VP3	235	N/A	62,179,180,181,234

	10D2	Common	9H2
VP1	90,113	88,89,106,107,108, 114, 226	87,91,102,103, 105,109,166,168, 227,228,282
VP2	N/A	137	138,139,141,171
VP3	N/A	N/A	233,235

	2E1	Common	9H2
VP1	97,98,99,104,214, 236	91,102,103,105, 107,168	87,88,89,100,101, 106,108,114,224, 228,229,240,281
VP2	N/A	N/A	137,140,141
VP3	183	N/A	235

	6B5	Common	9H2
VP1	99,109,214, 227,235	100,224,228,229	87,88,88,91,101, 102,103,105,106, 107,108,114,168, 240,281
VP2	138,139	137	140,141
VP3	182,183	N/A	235

Table S4: Paratope residues of FAb variable domain. Structural designation of paratope residues for FAb heavy and light chain variable domain (FR: Framing region, CDR: Complementarity determining region).

5E12 Variable Domain

Heavy Chain Residue	FAb region	Light Chain residue	FAb region
Q1	FR1	D28	CDR1
D54	CDR2	N36	CDR1
L56	CDR2	S65	FR3
F71	FR3	N66	FR3
D73	FR3	E68	FR3
R102	CDR3	T69	FR3
Y103	CDR3		
S105	CDR3		
N106	CDR3		
I107	CDR3		
R111	CDR3		

10D2 Variable Domain

Heavy Chain Residue	FAb region	Light Chain residue	FAb region
T108	CDR3	S27	CDR1
H109	CDR3	D113	CDR3
G110	CDR3		
W111	CDR3		

2E1 Variable Domain

Heavy Chain Residue	FAb region	Light Chain residue	FAb region
Y32	CDR1	A35	CDR1
W33	CDR1	E56	CDR2
Y52	CDR2	S83	CDR3
A103	CDR3		
S104	CDR3		
S105	CDR3		
L106	CDR3		
S109	CDR3		
S110	CDR3		

6B5 Variable Domain

Heavy Chain Residue	FAb region	Light Chain residue	FAb region
E23	CDR1	Y36	CDR1
S25	CDR1	S114	CDR3
D31	CDR1		
S56	CDR2		
S57	CDR2		
D59	CDR2		
S105	CDR3		
G106	CDR3		
G107	CDR3		
V108	CDR3		
L109	CDR3		
H110	CDR3		
Y112	CDR3		

Supplemental References:

1.Charnesky, A. J. *et al.* A human monoclonal antibody binds within the poliovirus receptor-binding site to neutralize all three serotypes. *Nat Commun* **14**, 6335 (2023).

2. Strauss, M. *et al.* Nectin-Like Interactions between Poliovirus and Its Receptor Trigger Conformational Changes Associated with Cell Entry. *J Virol* **89**, 4143–4157 (2015).

3. Miller, S. T., Hogle, J. M. & Filman, D. J. Ab initio phasing of high-symmetry macromolecular complexes: successful phasing of authentic poliovirus data to 3.0 A resolution. *J Mol Biol* **307**, 499–512 (2001).

4. Lentz, K. N. *et al.* Structure of poliovirus type 2 Lansing complexed with antiviral agent SCH48973: comparison of the structural and biological properties of the three poliovirus serotypes. *Structure* **5**, 961–978 (1997).

5. Bank, R. P. D. RCSB PDB - 1PVC: REFINEMENT OF THE SABIN STRAIN OF TYPE 3 POLIOVIRUS AT 2.4 ANGSTROMS AND THE CRYSTAL STRUCTURES OF ITS VARIANTS AT 2.9 ANGSTROMS RESOLUTION. https://www.rcsb.org/structure/1PVC.

6. Butan, C., Filman, D. J. & Hogle, J. M. Cryo-Electron Microscopy Reconstruction Shows Poliovirus 135S Particles Poised for Membrane Interaction and RNA Release. *J Virol* **88**, 1758–1770 (2014).

7. Xiao, C. & Rossmann, M. G. Interpretation of electron density with stereographic roadmap projections. *Journal of Structural Biology* **158**, 182–187 (2007).

8. Cui, Y. *et al.* Molecular basis of Coxsackievirus A10 entry using the two-in-one attachment and uncoating receptor KRM1. *Proceedings of the National Academy of Sciences* **117**, 18711–18718 (2020).

9.Zhou, D. *et al.* Unexpected mode of engagement between enterovirus 71 and its receptor SCARB2. *Nat Microbiol* **4**, 414–419 (2019).

10.Xu, L. *et al.* Cryo-EM structures reveal the molecular basis of receptor-initiated coxsackievirus uncoating. *Cell Host & Microbe* **29**, 448-462.e5 (2021).

11.Zhao, X. *et al.* Human Neonatal Fc Receptor Is the Cellular Uncoating Receptor for Enterovirus B. *Cell* **177**, 1553-1565.e16 (2019).

12. Baggen, J. *et al.* Role of enhanced receptor engagement in the evolution of a pandemic acute hemorrhagic conjunctivitis virus. *Proceedings of the National Academy of Sciences* **115**, 397–402 (2018).

13. Varanese, L. *et al.* MFSD6 is an entry receptor for enterovirus D68. *Nature* **641**, 1268–1275 (2025).

14 .Sievers, F. *et al.* Fast, scalable generation of high-quality protein multiple sequence alignments using Clustal Omega. *Mol Syst Biol* **7**, 539 (2011).

15. Liebschner, D. *et al.* Macromolecular structure determination using X-rays, neutrons and electrons: recent developments in Phenix. *Acta Cryst D* **75**, 861–877 (2019).

16. Pettersen, E. F. *et al.* UCSF Chimera--a visualization system for exploratory research and analysis. *J Comput Chem* **25**, 1605–1612 (2004).
